# Statement - COVID-19 Vaccination - Male and Female fertility, treatments to get pregnant, pregnancy

**DOI:** 10.5935/1518-0557.20220018

**Published:** 2022

REDLARA: Red Latinoamericana de Reproducción Asistida (Latin American Network of Assisted Reproduction)
SBRA: Associação Brasileira de Reprodução Assistida (Brazilian Association of Assisted Reproduction)
Pronúcleo: Associação Brasileira de Embriologistas em Medicina Reprodutiva (Brazilian Association of Embryologists in Reproductive Medicine)
ASPAMER: Asociación Panameña de Medicina Reproductiva (Panamanian Association of Reproductive Medicine)
SAMeR: Sociedad Argentina de Medicina Reproductiva (Argentinian Association of Reproductive Medicine)
AVEMERE: Asociación Venezolana de Medicina Reproductiva y Embriología (Venezuelan Association of Reproductive Medicine and Embryology)
SURH: Sociedad Uruguaya de Reproducción Humana (Uruguayan Society of Human Reproduction)
SBRH: Sociedade Brasileira de Reprodução Humana (Brazilian Society of Human Reproduction)
AMMR: Asociación Mexicana de Medicina Reproductiva (Mexican Association of Reproductive Medicine)
FPGO: Federación Paraguaya de Ginecología y Obstetricia (Paraguayan Federation of Gynaecology and Obstetrics)
AGFERH: Asociación Guatemalteca de Fertilidad y Reproducción Humana (Guatemalan Association of Fertility and Human Reproduction)

More than two years into the SARS-CoV-2 pandemic, the effects of the virus on the male and female reproductive system remain controversial. On the one hand, men with active infection harbor the virus for a short period, from 2 to 11 days (Guo *et al*., 2021; Holtmann *et al*., 2020), symptoms eventually referred to reproduction organs can be simply attributed to hyperthermia and/or or hypoxia. Infections in remission showed the virus in the semen in 1.4% versus 6% of the cases evaluated in the active phase, not seeming to worsen the spermogram parameters. A single case described as a positive semen test at 21 days of recovery showed that the stable partner, under unprotected sex, tested negative with oropharyngeal, rectal and vaginal smears (Gacci *et al*., 2021), indicating viral RNA and not a live virus active in the semen. In women, one can theoretically expect compromise of the ovary, uterus and vagina, as well as the endometrium and breasts, with menstrual disorders and secondary infertility (Jing *et al*., 2020), effects that have also not been clear to date (Li *et al*., 2021).

A variety of vaccines for SARS-CoV-2 have been developed, at record speed, thanks to the efforts of the world scientific community, associated with some government and industry efforts (Garg *et al*., 2021). However, the World Health Organization (WHO) has defined a situation of “vaccine hesitancy” (WHO, 2014), i.e., a delay or complete refusal to receive the vaccine, regardless of availability, with vaccines authorized through strict approval standards, with attenuated virus or m-RNA particles. Publications of false information that the incompletely inactivated virus or its particles have the potential ability to change the genetic information of an individual, could be introduced into the body, has negatively impacted patients who wish to become pregnant or even undergoing treatment to become pregnant, as well as pregnant women, lactating women and babies (Cha, 2021).

In this way, considering that:

1. More than 4 billion doses of vaccines have been given (Chen *et al*., 2021) to date, the CDC- Vaccine Adverse Event Reporting System, among more than 72 million Americans vaccinated, has recorded a small number of mild menstrual disorders (NIH, 2021).
2. Two recent studies demonstrate that vaccination had no influence on seminal parameters such as sperm concentration, seminal volume or sperm motility (Gonzalez *et al*., 2021; Safrai *et al*., 2022). Likewise, follicular steroidogenesis and oocyte quality did not differ between vaccinated and non-vaccinated individuals (Bentov *et al*., 2021).
3. Also, Orvieto *et al*. (2021) recording parameters of IVF cycles, also reported no difference between total and mature eggs retrieved, fertilization rates, and high-quality embryos. Aharon *et al*. (2022) complemented the information that nothing different was evidenced in relation to the rate of pregnancies and ongoing pregnancies, comparing vaccinated and non-vaccinated patients.
4. Preconception vaccination or vaccination before the 20^th^ week of pregnancy did not define a higher rate of spontaneous abortion in 2,456 pregnant women (Zauche *et al*., 2021).
5. There were no descriptions of placental compromise, perinatal or postnatal changes related to mRNA vaccines (Shanes *et al*., 2021), for women or their babies.
6. IgA levels were detected in nursing mothers at 2 weeks after vaccination, with IgG index increased at 4 weeks (one week after the second dose of mRNA vaccines), suggesting protection for the baby (Romero Ramírez *et al*., 2021).
7. The severity of COVID-19 illness, risk of death and adverse obstetric outcomes, and growing data on vaccine safety and effectiveness during pregnancy should outweigh any potential for individual adverse effects to the woman or her child.

The medical societies hereby represented recommend vaccination against the SARS-CoV-2 virus with the vaccine available, which should be encouraged with patients who are undergoing treatment to become pregnant, who are already pregnant, or even have recently given birth. To date, there is no evidence that the fertility potential of either men or women is affected by the use of vaccines.
